# “Bouncing temporomandibular joint”: Multiple temporomandibular lesions in a patient with rheumatoid arthritis

**DOI:** 10.1515/rir-2024-0033

**Published:** 2025-01-09

**Authors:** Jun Li, Xin Gao, Haitao Dong, Jiuliang Zhao

**Affiliations:** Department of Rheumatology and Clinical Immunology, Peking Union Medical College Hospital (PUMCH), Chinese Academy of Medical Sciences & Peking Union Medical College, Beijing 100730, China; Department of Radiology, Peking Union Medical College Hospital (PUMCH), Chinese Academy of Medical Sciences & Peking Union Medical College, Beijing 100730, China; Department of Stomatology, Peking Union Medical College Hospital (PUMCH), Chinese Academy of Medical Sciences & Peking Union Medical College, Beijing 100730, China; State Key Laboratory of Complex Severe and Rare Diseases, Beijing 100730, China; National Clinical Research Center for Dermatologic and Immunologic Diseases (NCRC-DID), Ministry of Science & Technology, Beijing 100730, China; Key Laboratory of Rheumatology and Clinical Immunology, Ministry of Education, Beijing 100730, China

**Figure 1:**
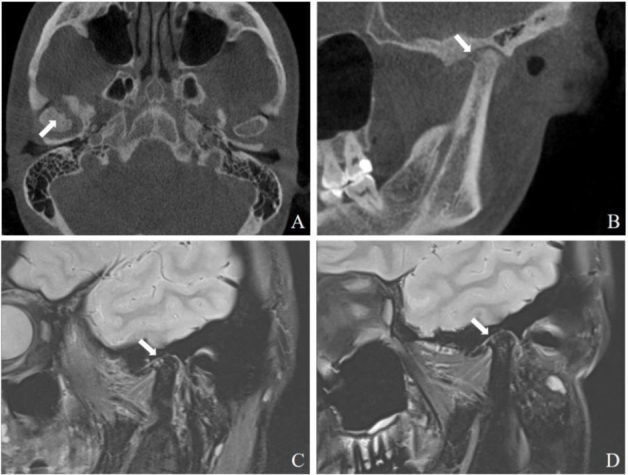
A: Axial view of CBCT shows osteophyte and surface erosion in the anterior bevel of the right condyle (arrow). B: Oblique sagittal view of CBCT shows osteophyte and surface erosion in the anterior bevel of the right condyle (arrow). C: MRI shows subcortical cyst in the right joint fossa and condyle (arrow). D: MRI shows reduction of cystic lesions in the right articular fossa and condyle after treatment (arrow).

A 39-year-old female was admitted due to bilateral temporomandibular joint (TMJ) pain, more severe on the left side than the left dise, accompanied by a clicking sound for 3 months. These symptoms were more noticeable during eating, occasionally leading to chewing difficulties. The patient denied any history of trauma or gum disease and reported no discomfort in other joints. She was positive for high titers of anticyclic citrullinated peptide (CCP) antibodies ( > 200 U/mL) and anti-mutant citrullinated vimentin (MCV) antibodies ( > 1000 U/mL). Her rheumatoid factor (RF), erythrocyte sedimentation rate (ESR), and C-reactive protein (CRP) levels were all within the normal range. The cone-beam computed tomography (CBCT) of TMJ showed osteophyte and surface erosion of the anterior bevel of the right condyle (Figure 1 A-B). Magnetic resonance imaging (MRI) of the TMJ revealed subcortical cyst at the anterior bevel of the right condyle, along with deformities of both articular discs and irreducible anterior internal displacement (Figure 1C). She was given a probable diagnosis of rheumatoid arthritis (RA) and was initially treated with methotrexate (15 mg/wk). Six months later, her TMJ pain and clicking sound improved significantly. MRI showed reduced cystic degeneration of the right condyle compared to previous scans (Figure 1D).

Involvement of the TMJ in patients with RA is common, ranging between 45% and 92%.^[1]^ However, temporomandibular disorder (TMD) related to RA is underestimated, largely due to its non-specific clinical manifestations and was not routinely examed.^[2,3]^ MRI is currently the gold standard for diagnosing TMD because of its high resolution to differentiate joint and its surrounding soft tissue. CBCT can clearly display bone details. Therefore, MRI combined with CBCT can provide a more comprehensive assessment of TMD in patients with RA. TMJ involvement in RA can cause abnormalities in bone, joint space, and surrounding soft tissue, resulting in diverse clinical manifestations.^[1]^ The destruction or absorption of condylar bone and cysts of the condyle may be specific imaging features of RA-TMJ, helping in distinguishing from other conditions like osteoarthritis (OA).^[4,5]^
